# Guanylin and uroguanylin are produced by mouse intestinal epithelial cells of columnar and secretory lineage

**DOI:** 10.1007/s00418-016-1453-4

**Published:** 2016-05-31

**Authors:** Pauline T. Ikpa, Hein F. B. M. Sleddens, Kris A. Steinbrecher, Maikel P. Peppelenbosch, Hugo R. de Jonge, Ron Smits, Marcel J. C. Bijvelds

**Affiliations:** 1 Department of Gastroenterology and Hepatology, Erasmus MC University Medical Center, PO Box 2040, 3000 CA Rotterdam, The Netherlands; 2 Department of Pathology, Erasmus MC University Medical Center, PO Box 2040, 3000 CA Rotterdam, The Netherlands; 3 Division of Gastroenterology, Hepatology and Nutrition, Cincinnati Children’s Hospital Medical Center, Cincinnati, OH 45229 USA

**Keywords:** Cyclic guanosine monophosphate, Enteroendocrine cells, Guanylyl cyclase C, Intestinal fluid transport

## Abstract

Guanylin (GN) and uroguanylin (UGN), through activation of guanylyl cyclase C (GCC), serve to control intestinal fluid homeostasis. Both peptides are produced in the intestinal epithelium, but their cellular origin has not been fully charted. Using quantitative PCR and an improved in situ hybridization technique (RNAscope), we have assessed the expression of GN (*Guca2a*), UGN (*Guca2b*), and GCC (*Gucy2c*) in mouse intestine. In the crypts of Lieberkühn, expression of *Guca2a* and *Guca2b* was restricted to cells of secretory lineage, at the crypt’s base, and to a region above, previously identified as a common origin of cellular differentiation. In this compartment, comparatively uniform levels of *Guca2a* and *Guca2b* expression were observed throughout the length of the gut. In contrast, *Guca2a* and *Guca2b* expression in the villus–surface region was more variable, and reflected the distinct, but overlapping expression pattern observed previously. Accordingly, in jejunum and ileum, *Guca2a* and *Guca2b* were abundantly expressed by enterocytes, whereas in colon only *Guca2a* transcript was found in the surface region. In duodenum, only low levels of *Guca2b* transcript were observed in columnar cells, and *Guca2a* expression was restricted entirely to cells of the secretory lineage. *Gucy2c* was shown to be expressed relatively uniformly along the rostrocaudal and crypt–villus axes and was also found in the duodenal glands. Our study reveals novel aspects of the cellular localization of the GCC signaling axis that, apart from its role in the regulation of fluid balance, link it to pH regulation, cell cycle control, and host defense.

**Electronic supplementary material:**

The online version of this article (doi:10.1007/s00418-016-1453-4) contains supplementary material, which is available to authorized users.

## Introduction

Signal transduction through the receptor–enzyme guanylyl cyclase C (GCC) serves to control intestinal fluid balance. Activation of the luminal, extracellular receptor domain by one of two locally produced peptides, guanylin (GN) and uroguanylin (UGN), leads to a surge in cellular cGMP levels, which prompts osmotic water transport to the intestinal lumen by protein kinase-mediated stimulation of anion secretion through the cystic fibrosis transmembrane conductance regulator (CFTR) anion channel and inhibition of sodium absorption through the sodium-proton exchanger type 3 (NHE3) (Vaandrager 2002). This pivotal role of GCC signaling in intestinal fluid balance is most poignantly illustrated by gain- and loss-of-function mutations in *GUCY2C* (encoding GCC), which have been shown to cause secretory diarrhea and intestinal obstruction, respectively (Romi et al. 2012; Fiskerstrand et al. 2012; Muller et al. 2015). Furthermore, GCC is activated by the (U)GN mimetic heat-stable toxin (STa) produced by enterotoxigenic *Escherichia coli*, a frequent cause of infectious diarrhea.

Apart from its role in the regulation of fluid homeostasis, GCC signaling may also regulate luminal pH. GCC stimulation strongly enhances duodenal bicarbonate secretion, and some data suggest that luminal acid may stimulate UGN release from duodenal enteroendocrine cells (Guba et al. 1996; Bengtsson et al. 2007; Kokrashvili et al. 2009; Joo et al. 1998; Rao et al. 2004; Singh et al. 2008). Evidently, in proximal intestine, such a feedback loop may aid the neutralization of the acid load entering from the stomach. In addition, it has become apparent that local bicarbonate production is crucial for the proper expansion of the mucins produced by goblet cells, and, consequently, the physiochemical properties of the mucus layer covering the epithelium along the entire tract (Garcia et al. 2009; Gustafsson et al. 2012). Possibly connected to its effect on mucin unfolding and fluid transport, loss of GCC signaling was shown to impair epithelial barrier function and innate host responses to bacterial pathogens (Mann et al. 2013; Han et al. 2011).

Lastly, GCC signaling may modulate the cell cycle in small intestinal and colonic epithelium. Mice deficient in either GN-, GCC-, or the cGMP-dependent protein kinase operating downstream of GCC (PRKG2), exhibit enhanced cell proliferation in the crypts and a reduction in the amount of cells of the secretory lineage (Li et al. 2007a; Steinbrecher et al. 2002; Wang et al. 2012b). It has also been suggested that loss of GCC signaling predisposes to tumor development (Li et al. 2007b).

The convergence of such apparently disparate physiological processes at the level of GCC suggests a strict compartmentalization of signaling routes. In this context, it is of interest that various distinct cell types within the intestinal epithelium have been proposed to produce GN and UGN. Mostly, these studies indicate production by cells from the secretory (granulocytic) lineage. In human small intestine, GN transcript was localized to the Paneth cells, whereas in the rat, GN transcript and protein were detected in goblet cells, and UGN transcript was found in enterochromaffin (EC) cells (De Sauvage et al. 1992; Cohen et al. 1995; Perkins et al. 1997; Date et al. 1996). In rat colon, GN was expressed by goblet cells, but also by the columnar cells of the surface epithelium, whereas in guinea pig, GN immunoreactivity was restricted to EC cells (Cohen et al. 1995; Cetin et al. 1994; Li et al. 1995). Finally, in mouse intestine, UGN transcript was localized exclusively to the villus, whereas GN transcript was found in both the villi and crypts of the small intestine and in the surface epithelium of colon (Whitaker et al. 1997). These disparate results may signify marked differences in the localization, and, perhaps, the physiological role of GN and UGN between species, and different anatomical regions. In addition, they may reflect the technical limitations of classical in situ hybridization and immunological methods.

Our study aimed to ascertain the expression pattern of GN, UGN, and GCC, using quantitative PCR and an improved in situ hybridization technique, which drastically improves sensitivity. While our study, in the main, corroborates previously reported patterns of GN, UGN, and GCC partitioning along the rostrocaudal axis of the intestine, these technical advances have allowed us to chart these patterns in more detail, revealing previously unappreciated differences in the expression pattern of duodenum versus jejunum, and the cell lineages involved in the production of GN, UGN, and GCC.

## Materials and methods

### Animals

Mice (FVB) were maintained in an environmentally controlled facility at the Erasmus MC, Rotterdam. All experiments were performed on animals, 8–16 weeks of age, and were approved of by the Ethical Committee for Animal Experiments of the Erasmus MC.

### Tissue collection

Mice were anaesthetized (ketamine 100 mg/kg, xylazine 20 mg/kg; i.p.), and the intestinal tract was collected and flushed with ice-cold saline. All tissue was harvested between 12:00 and 14:00 h, to control for diurnal variations in gene expression.

For assessing the partitioning of transcript along the rostrocaudal axis of the intestinal tract, 6 equidistant segments (length: 0.5 cm) of small intestine and 2 segments of the colon were excised. The small intestine was sampled, starting 1 cm caudal to the pyloric sphincter and up to 1 cm proximal to the ileocecal valve. Colon was sampled at 1/3 and 2/3 of its entire length. Tissue was flash frozen in liquid nitrogen and stored at −80 °C.

For RNAscope analysis and lectin UEA1 (*Ulex europaeus* agglutinin 1) staining (see below), the excised intestine was flushed with ice-cold saline, cut open lengthwise, rolled into a Swiss roll, and immersed in PBS-buffered formalin (10 %) for 24 h at 4 °C. After fixation, tissue was embedded in paraffin, according to established protocols.

### Quantitative polymerase chain reaction (qPCR)

Tissue was homogenized with a rotor–stator homogenizer in TRIzol reagent (Qiagen), and total RNA was extracted using the NucleoSpin RNA kit (Macherey–Nagel). After the integrity of the extracted RNA was verified by gel electrophoresis, cDNA was synthesized using the PrimeScript RT master mix (Takara Bio).

Quantitative PCR (primer sequences shown in Table S1) was performed on cDNA, using the SYBR Select Master Mix (Applied Bio System). Median values from assays performed in triplicate were used to determine the expression levels of *Guca2a*, *Guca2b,* and *Gucy2c*, relative to *Gapdh*.

### In situ hybridization by RNAscope

Probes for detection of murine GN, UGN, and GCC transcripts were purchased from Advanced Cell Diagnostics (product code: 427996, 428006, 436596, respectively). Because, other than in conventional in situ hybridization, multiple independent probes have to hybridize to the target sequence in tandem in order for signal amplification to occur, RNAscope ensures selective amplification of target-specific signals (Wang et al. 2012a). RNAscope was performed according to the instructions of the manufacturer of the probes and the reagent kit (VS Reagent Kit 320600; Advanced Cell Diagnostics), on proteinase K (0.1 %, 5 min at 37 °C)-treated paraffin sections (5 µm). Detection of the ubiquitously expressed gene peptidylprolyl isomerase B (*Ppib*) served to ensure that tissue sections were correctly primed for probe hybridization (Advanced Cell Diagnostics, product code: 313919).

### Histochemical detection of fucose glycoprotein

For detection of fucose glycoprotein-producing cells, sections used for RNAscope were re-hydrated in PBS, treated with a biotin blocking reagent (Dako), and incubated (1 h, at room temperature) with biotinylated lectin UEA1 (Vector Labs). Lectin UEA1-labeled carbohydrate moieties were visualized with a streptavidin-conjugated fluorescent probe (Alexa Fluor 594; Life Technologies). In negative controls, incubation with lectin UEA1 was omitted. Slides were mounted with ProLong Gold antifade reagent (Life Technologies) and stored at 4 °C.

## Results

### Partitioning of *Guca2a*, *Guca2b*, and *Gucy2c* transcripts along the rostrocaudal axis of the mouse intestinal tract

**Fig. 1:**
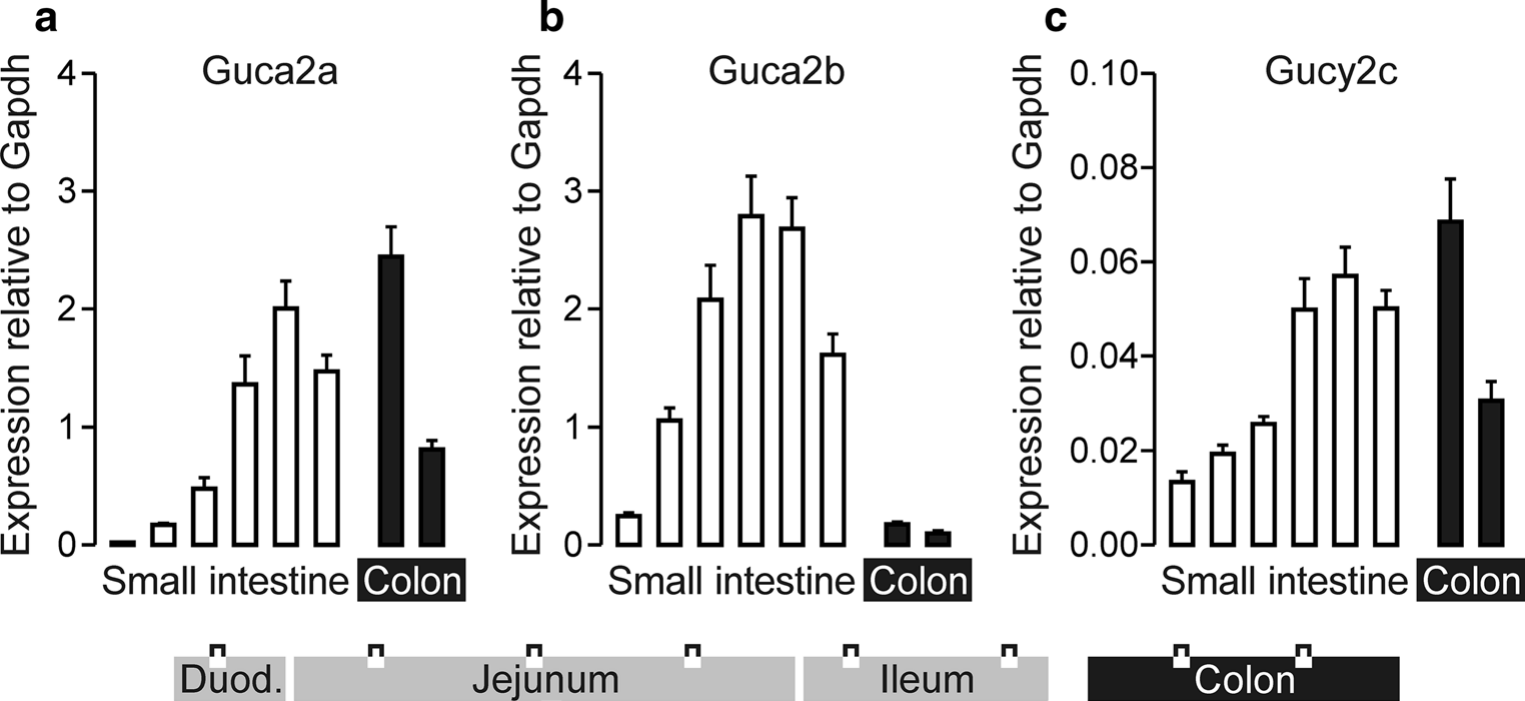
Partitioning of *Guca2a* (**a**), *Guca2b* (**b**), and *Gucy2c* (**c**) transcripts along the rostrocaudal axis of the mouse intestinal tract. Transcript levels in 6 equidistant sections of small intestine and 2 sections of colon (see diagram) were assessed by qPCR, using expression of *Gapdh* as a reference. Data depict mean ± standard error. *N* = 6

Quantitative PCR analysis showed that *Guca2a* transcript levels gradually increased along the rostrocaudal axis of the small intestine, and peaked in the proximal colon (Fig. 1a). In contrast, *Guca2b* transcript levels were low in colon. *Guca2b* levels were also low in the duodenum, but rose steeply along the rostrocaudal axis, and peaked in the middle to distal part of the small intestine (Fig. 1b). *Gucy2c* was expressed at much lower (>tenfold) levels than *Guca2a* or *Guca2b* and was partitioned more uniformly (Fig. 1c). Distribution of these transcripts was similar in male and female mice (not shown).

**Fig. 2:**
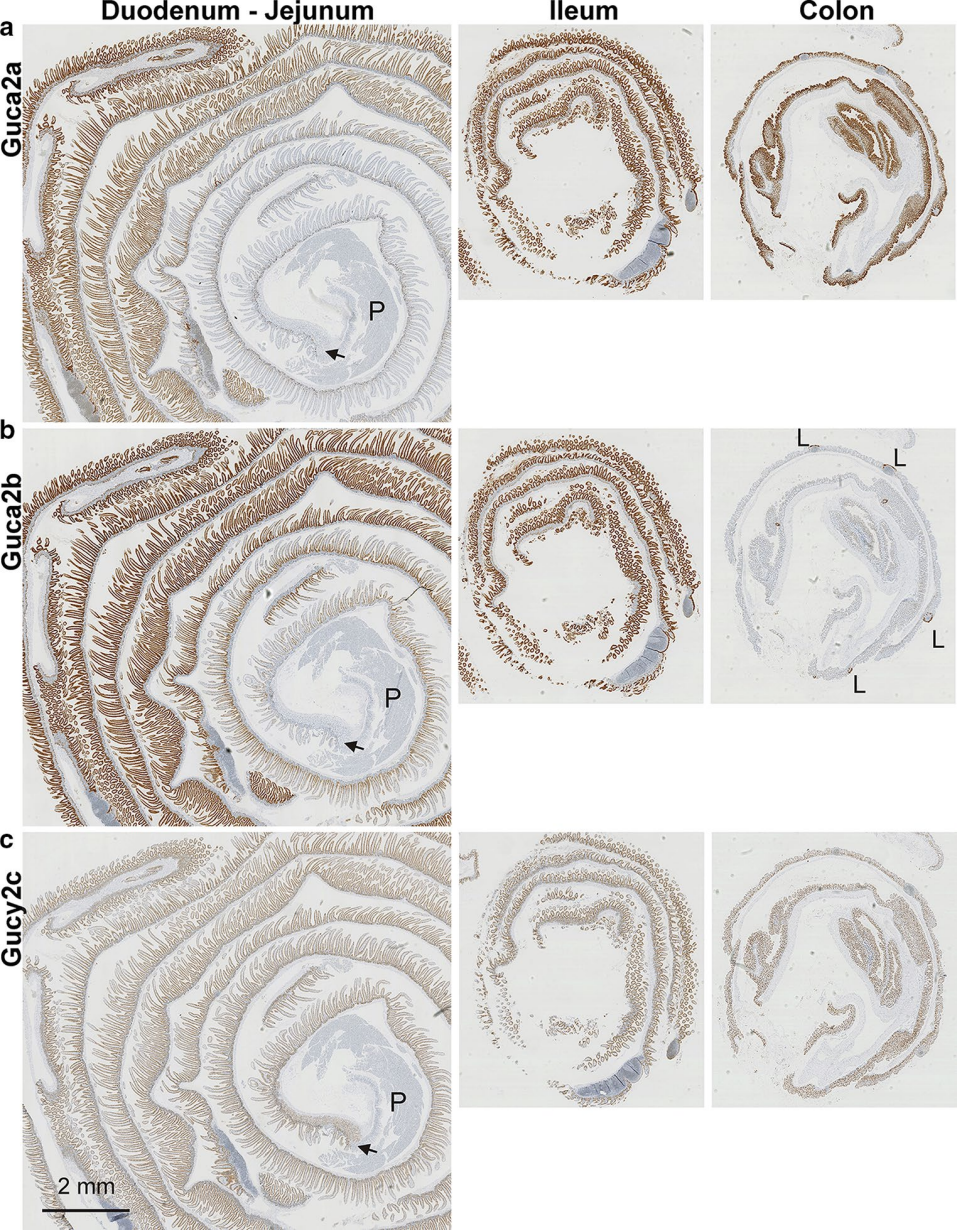
Partitioning of *Guca2a* (**a**), *Guca2b* (**b**), and *Gucy2c* (**c**) transcripts in intestinal mucosa. Intestinal tissue was paraffin-embedded in a “Swiss roll” configuration and was probed by RNAscope. The *arrow* indicates the transition from gastric to duodenal epithelium. *P* pancreatic tissue, *L* lymphoid tissue

The RNAscope technique was used to visualize *Guca2a*, *Guca2b,* and *Gucy2c* transcripts in longitudinal sections of mouse intestine. This methodology employs up to 20 pairs of oligonucleotide probes per transcript, of which the paired probes need to hybridize in close proximity in order for signal amplification to occur. As a consequence, this technique provides a strongly improved signal-to-noise ratio compared with conventional in situ hybridization techniques (Wang et al. 2012a). The distribution pattern that emerged for these transcripts closely matched the expression profile assessed by qPCR analysis, i.e., the gradual increase of *Guca2a* and *Guca2b* expression from duodenum to the distal small intestine, the high expression of *Guca2a*, but low expression of *Guca2b*, in colon, and the comparatively low expression of *Gucy2c* (Fig. 2). This agreement strongly suggests specific hybridization of the probes. In addition, probe hybridization was restricted to intestinal epithelial cells, i.e., absent from underlying connective and muscle tissue, consistent with the discrete epithelial expression pattern of these genes. Our data also corroborate the previously observed high levels of focal *Guca2b* expression in regions of the colonic epithelium that border on lymphoid tissue (Fig. 2b colon section, Fig. 4j) (Whitaker et al. 1997). Specificity of the *Guca2a* probes was further corroborated by RNAscope performed on intestinal tissue of *Guca2a* null mice, in which only sparse punctuate staining was found in the nuclei (indicating weak hybridization with DNA) and the cytoplasmic region (indicating weak hybridization with truncated *Guca2a* transcripts; Fig. S1).

The observed rostrocaudal distribution patterns of GN, UGN, and GCC are congruent with those previously reported for rat intestine (Qian et al. 2000), but differ somewhat from those reported in a previous mouse study, in which the levels of *Guca2b* expression in duodenum and jejunum were shown to be similar (Whitaker et al. 1997). However, in this previous study, tissue from the proximal 3 cm of the mouse small intestine was pooled to assess duodenal expression, whereas we used a section between 1 and 1.5 cm distal to the pylorus. As is apparent from the RNAscope data (Fig. 2b), the expression level of *Guca2b* steeply increases within the first few centimeters of the small intestine, indicating that these apparent differences may be due largely to the different sampling strategies. Furthermore, it is conceivable that these differences reflect the effect of an unidentified environmental factor (i.e., diurnal rhythm, food intake, and composition) on duodenal *Guca2b* expression.

**Fig. 3:**
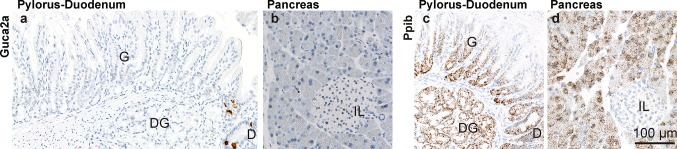
Partitioning of *Guca2a* and *Ppib* transcripts in the pyloric–duodenal region and in pancreas. **a**, **b** *Guca2a* expression was found in the duodenum (D), in cells at the base of the crypts of Lieberkühn, but was not detected in the gastric (G) or the duodenal glands (DG), or in the pancreas. **c**, **d** Detection of *Ppib* transcript in glandular epithelia in the pyloric–duodenal region and pancreas. *IL* islet of Langerhans

We did not detect *Guca2a* transcript in gastric or in pancreatic epithelia (Figs. 2a, 3), nor did we detect expression of *Guca2b* or *Gucy2c* in these tissues (Fig. 2b, c). This contrasts with previous observations on guinea pig, in which GN immunoreactivity was observed in pyloric EC cells (Cetin et al. 1994), and with observations on human and rat pancreatic tissue, in which GN, UGN, and GCC transcript and protein were detected (Kulaksiz et al. 2001; Kulaksiz and Cetin 2002). RT-PCR analysis confirmed that, compared to the gut, these glandular tissues contain only very low levels of these three transcripts (not shown). Such transcripts of low abundance may not be detectable by in situ hybridization, particularly in the pancreas, which contains high levels of ribonucleases (Azevedo-Pouly et al. 2014). However, arguing against significant mRNA loss prior to tissue fixation, we could readily detect *Ppib* transcript, in both the stomach and the pancreas (Fig. 3c, d). Therefore, we tentatively conclude that expression of the GCC signaling axis is low in mouse, compared to human pancreas. Interestingly, this apparent difference in the pancreatic expression of the GCC signaling axis between these species mirrors a previously observed disparity in the regulation of ductal anion and fluid secretion: Whereas in the human pancreas, the GCC signaling axis is thought to control the activity of the phosphorylation-regulated CFTR anion channel, anion and fluid secretion in the murine exocrine pancreas is principally governed by Ca^2+^-dependent anion channels, which operate independently of the GCC pathway (Winpenny et al. 1995; Kulaksiz et al. 2001).

### Partitioning of *Guca2a* and *Guca2b* transcripts along the crypt–villus and crypt–surface axis of the mouse small intestine and colon, respectively

**Fig. 4:**
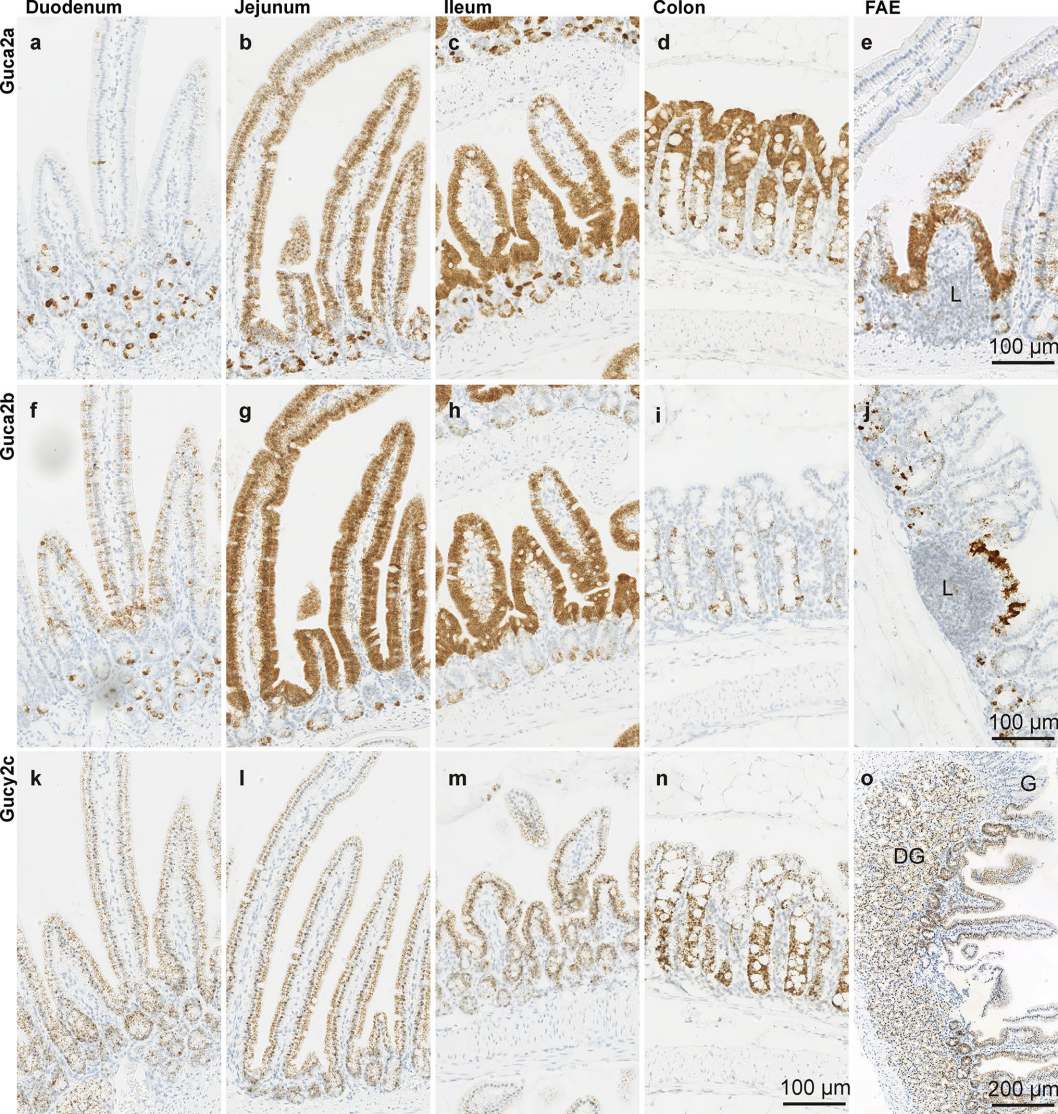
Localization of *Guca2a* (**a**–**d**), *Guca2b* (**f**–**i**), *and Gucy2c* (**k**–**n**) transcripts in intestinal epithelium. **e** *Guca2a* expression in follicle-associated epithelium in proximal jejunum. **j** *Guca2b* expression in follicle-associated epithelium in colon. **o** *Gucy2c* expression in the region of transition from the gastric (G) to duodenal mucosa, with duodenal glands (DG) below. *L* lymphoid tissue

Along the entire length of the small intestine, high *Guca2a* transcript levels were found in cell clusters at the base of the crypts and in solitary cells that line the lateral sides of the crypts (Fig. 4a–d). In the proximal small intestine, expression in the villus region was restricted to a distinct, sparsely distributed cell type (Fig. 4a). Very little expression was found in columnar, absorptive villus cells (enterocytes). However, in the proximal jejunum, expression of *Guca2a* in the villus became more prominent and was found in most columnar cells. *Guca2a* expression in the villus region further increased along the rostrocaudal axis, and distal jejunum and ileum showed abundant *Guca2a* expression in enterocytes (Fig. 4b, c). In colon, *Guca2a* transcript was found along the entire crypt–surface axis, but appeared to be most abundantly expressed by cells lining the lateral sides of the crypts and the surface. Strong expression was also found in cell clusters at the base of the crypts (Fig. 4d). *Guca2a* was also strongly expressed by the follicle-associated epithelium overlying lymphoid aggregates, in both small and large intestine (Fig. 4e).

Like for *Guca2a*, *Guca2b* transcript was found in cells at the base and the lateral sides of the crypts, along the length of the small intestine (Fig. 4f–h). However, the level of cellular expression, and the amount of cells that expressed *Guca2b* in the crypts, appeared somewhat lower than for *Guca2a*. In the duodenum, *Guca2b* expression started to emerge at the base of the villi and involved the entire height of the villus in more distal sections of the small intestine (Fig. 4f–h). In colon, *Guca2b* expression was restricted to individual cells, or small clusters of cells, in the crypts (Fig. 4i). Although small in number, the level of expression per single cell was relatively high and comparable to the level in *Guca2b*-expressing cells in the crypts of the small intestine. As for *Guca2a*, we observed strong *Guca2b* expression in the follicle-associated epithelium overlying lymphoid tissue, in both small and large intestine (Fig. 2b colon section, Fig. 4j).

### Partitioning of Gucy2c transcript along the crypt–villus and crypt–surface axis of the mouse small intestine and colon, respectively

*Gucy2c* was expressed relatively uniformly, in both the crypt and villus region of the epithelium (Fig. 4k–n). As for *Guca2a* and *Guca2b*, *Gucy2c* probe hybridization was restricted to intestinal epithelial cells. Expression in these cells was relatively uniform; only at the tip of the villi, expression seemed to wane slightly (similar was observed for *Guca2a* and *Guca2b*). No specific subset of cells was shown to be enriched in (or depleted of) *Gucy2c* transcript.

Further, we found *Gucy2c* transcripts in the duodenal glands (Fig. 4o). Previously, by autoradiographic demonstration of STa-specific binding sites in the intestinal mucosa, the presence of GCC in the duodenal glands of the North American opossum had been inferred (Krause et al. 1990). However, similar studies performed on human duodenal mucosa failed to demonstrate specific STa binding (Krause et al. 1994). Remarkably, the duodenal glands did not contain *Guca2a* or *Guca2b* transcript, indicating that local GCC activation relies on GN or UGN produced elsewhere.

### Localization of Guca2a and Guca2b in cells of secretory lineage

**Fig. 5:**
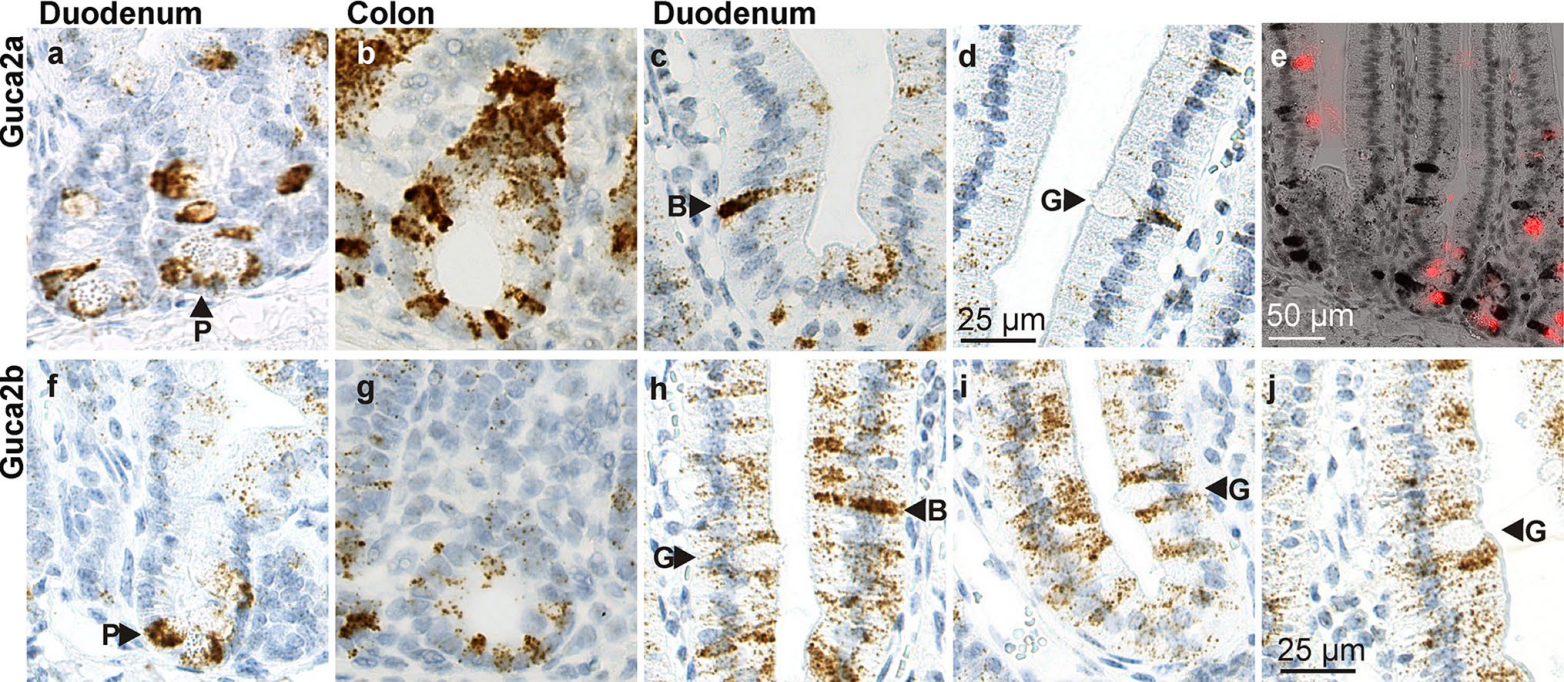
*Guca2a* and *Guca2b* expression by cells of the secretory lineage in the duodenum and colon. **a** *Guca2a* expression in Paneth cells in duodenum. **b** *Guca2a* expression in cells at the base and the neck region of the crypts in colon. **c** *Guca2a* expression in a duodenal brush cell. **d** *Guca2a* expression in a duodenal goblet cell. **e** Costaining of *Guca2a* transcript (*dark brown*) and lectin UEA1-binding fucose glycoproteins (*bright red*) in duodenum. **f** *Guca2b* expression in Paneth cells in duodenum. **g** *Guca2b* expression in cells at the base of the crypts in colon. **h** *Guca2b* expression in a duodenal brush cell. No *Guca2b* transcript was observed in goblet cells. **i**, **j** *Guca2b* levels were generally low in columnar cells, but comparatively high in columnar cells adjoining goblet cells. *B* brush cell, *G* goblet cell, *P* Paneth cell

In the small intestine, the *Guca2a-* and *Guca2b*-expressing cells at the base of the crypt were found to contain secretory granules and (in part) colocalized with Paneth cells (Fig. 5a, f). In contrast, *Guca2a* or *Guca2b* expression was not observed in the adjacent stem cells. A previous in situ hybridization study suggested that apparent *Guca2b* localization at the base of the crypts was a result of autofluorescence of the Paneth cells (Whitaker et al. 1997), but since RNAscope uses an enzymatic detection method, this cannot have confounded our observations. Moreover, our detection of *Guca2a* and *Guca2b* in Paneth cells is consistent with studies using a fluorescence-activated cell sorting technique to isolate specific epithelial cell lineages (Sato et al. 2011; Cash et al. 2006). This showed that *Guca2a* and *Guca2b* rank in the 90th percentile of most highly expressed genes in murine Paneth cells, and that Paneth cells are highly enriched in these transcripts, relative to stem cells (NCBI GEO datasets GSE5156 and GSE25109). In addition, these datasets reveal that *Gucy2c* is expressed at near-equal levels in Paneth and stem cells, congruent with the relatively uniform distribution of *Gucy2c* transcript in crypt epithelium (Fig. 4k–n).

*Guca2a* and *Guca2b* transcripts were also found in cells at the base of the crypts in colon (Fig. 5b, g). These cells appear to occupy the same niche as the Paneth cells of the small intestine, suggesting they represent the cKit-positive Paneth-like cells identified previously (Rothenberg et al. 2012). In earlier studies, only *Guca2a* transcript had been detected in this compartment, possibly because the low sensitivity of conventional in situ hybridization techniques precluded detection of the less abundant *Guca2b* transcripts (Whitaker et al. 1997).

Lectin UEA1 staining confirmed that the cells at the base of the crypts and other *Guca2a*- and *Guca2b*-positive cells in the crypts and villus region of duodenum were of secretory lineage (Fig. 5e). Based on their numbers, localization, and typical shape, these *Guca2a-* and *Guca2b*-expressing cells in the villus appear to include brush (tuft) cells (Fig. 5c, h). Previously, a murine duodenal enteroendocrine cell-type secreting β-endorphin in response to acidic or hyperosmotic stimulation was identified (Kokrashvili et al. 2009). Interestingly, this cell type also contained UGN (but, based on immunological detection methods, not GN). In rat, apart from goblet cells, some enteroendocrine cell types also seemed to contain immunoreactive GN, although the specificity of the signal was questioned by the authors (Li et al. 1995). Collectively, these data strongly suggest that distinct brush cell populations coexist in the murine duodenal epithelium, expressing *Guca2a* or *Guca2b*.

In addition, *Guca2a* expression was found in a subset of goblet cells in the lower villus (Fig. 5d). This suggests that GN may be released concurrently with the mucin-containing granules produced by goblet cells. No *Guca2b* was found in goblet cells, but in the duodenum, where *Guca2b* expression is generally low, columnar cells that adjoined goblet cells seemed to have relatively (compared to more remote columnar cells) high *Guca2b* expression, suggesting that UGN release from these cells may play a role in goblet cell function (Fig. 5h–j). These findings are in line with studies on rat, which detected GN, but not UGN, in a subset of goblet cells of the rat small intestine and colon (Li et al. 1995; Cohen et al. 1995).

## Discussion

An improved in situ hybridization technique (RNAscope) has allowed us to reveal novel aspects of the localization of the GCC signaling axis in the murine intestinal tract. We demonstrate strong expression of *Guca2a* and *Guca2b* transcripts in cells of secretory lineage, in particular Paneth cells located at the base of the crypts and chemosensory brush cells in the lower villus. Strikingly, the general pattern of *Guca2a* and *Guca2b* expression in this lineage is relatively uniform along the entire intestine: Even in the proximal part, which was thought to produce little GN, we observed prominent *Guca2a* expression in Paneth and brush cells, whereas in colon, which was thought to produce little UGN, robust *Guca2b* expression was found in Paneth-like cells at the base of the crypts.

In contrast to this relatively stable expression pattern in the crypts, expression in the villus–surface compartment showed more variation between anatomical regions, as expression of *Guca2a* and *Guca2b* extends to cells from the columnar (absorptive) lineage. It is chiefly the expression in this compartment that reiterates the previously reported pattern of GN and UGN distribution, i.e., a predominance of UGN in the proximal gut, and of GN in the colon (Qian et al. 2000; Whitaker et al. 1997). As has been noted before this pattern is consistent with the observation that GN is a relatively (in comparison with UGN) poor GCC ligand at the low pH prevailing in the proximal small intestine (Whitaker et al. 1997). Nevertheless, prominent expression of *Guca2a* is found in the crypts of the proximal small intestine, suggesting that the pH in this area may be maintained at more neutral values (aided by local bicarbonate secretion).

Along the length of the small intestine, *Guca2a* and *Guca2b* transcripts were found in Paneth cells, or, in case of the colon, in cells which appear to occupy the same position as these. *Guca2a* expression appeared more pronounced than *Guca2b*, but the localization pattern was similar. Previous in situ hybridization studies showed a similar distribution of *Guca2a* at the base of the crypts in jejunum and ileum of mice, although the exact cell types involved could not be ascertained (Whitaker et al. 1997). *Guca2a* and *Guca2b* expression by murine Paneth cells was also indicated in studies that employed cell sorting techniques to isolate epithelial cells representing different lineages (Sato et al. 2011; Cash et al. 2006). Furthermore, GN transcript was localized to human Paneth cells (De Sauvage et al. 1992).

It has been suggested that GN-stimulated fluid secretion emanating from the lower crypt may have a cleansing effect that supports the action of the bactericidal products of the Paneth cells (De Sauvage et al. 1992). In further support of innate immunity, GN and/or UGN release from Paneth cells may trigger bicarbonate secretion from neighboring CFTR-containing columnar cells, facilitating expansion of condensed mucins, and, consequently, the formation of the protective mucus layer covering the epithelium (Gustafsson et al. 2012; Garcia et al. 2009). In accordance with this notion, it has been shown that GCC signaling reduces susceptibility to infection by attaching/effacing bacterial pathogens (Mann et al. 2013). GN stored in goblet cells (and UGN in adjoining columnar cells) may serve a similar role. When released together with the mucin granules, a synchronized activation of GCC-dependent bicarbonate secretion from adjacent enterocytes (as goblet cells seem to contain little CFTR) may aid the proper expansion of the secreted condensed mucin granules. Consistent with this hypothesis, exocytosis from goblet cells is under cholinergic control, and it has been shown that cholinergic input also stimulates GN release in rat colon (Martin et al. 1999; Phillips 1992).

Apart from producing antibacterial products, Paneth cells also produce various factors that modulate stem cell function. It is conceivable that GN and UGN may also contribute to this modulation. In mice, it has been shown that GN, PRKG2, or GCC deficiency leads to enhanced crypt cell proliferation, and that GCC deficiency reduces goblet and Paneth cell numbers in mouse small intestine, whereas PRKG2 deficiency reduces goblet cell numbers in colon (Li et al. 2007a; Steinbrecher et al. 2002; Wang et al. 2012b). These results suggest that GCC signaling directs differentiation to a secretory lineage. Indeed, PRKG2 has been shown to control the activity of the transcription factor Sox9, which is a key determinant of Paneth cell formation (Mori-Akiyama et al. 2007; Swartling et al. 2009; Chikuda et al. 2004). Because of their close contact to stem cells and their progenitors, the Paneth cells are a likely source of the ligands that control GCC activity in this niche. Consistent with this model, both our present data and previous studies (NCBI GEO datasets GSE5156 and GSE25109) indicate that *Gucy2c* expression in the crypts extends to the stem cells, whereas Guca2a and Guca2b expression is restricted to cells of the secretory lineage (Sato et al. 2011; Cash et al. 2006).

In duodenum, prominent expression of *Guca2a* and *Guca2b* in the villi is mostly confined to just the few cells of secretory lineage. Apart from Paneth and goblet cells, this includes brush cells. UGN, but not GN, has previously been localized to murine brush cells (Kokrashvili et al. 2009). Brush cells are known to monitor the composition of the lumen, and it was shown that this particular subset of UGN-containing duodenal brush cells secretes β-endorphin into the lumen upon acidic or hypertonic stimulation (Kokrashvili et al. 2009; Ronnestad et al. 2014). We propose that the UGN stored in this cell type may also be released upon exposure to these luminal stimuli. In support, an increase in bicarbonate secretion was observed in mouse duodenum upon lowering of the luminal pH, which may be attributed to endogenous (U)GN release, as GN and UGN are known to strongly stimulate duodenal bicarbonate secretion (Singh et al. 2008; Guba et al. 1996; Joo et al. 1998). This assumption is also consistent with the observation that (I) luminal hypertonicity stimulates the release of GN and UGN and inhibits NHE3-mediated sodium absorption (Gawenis et al. 2003; Steinbrecher et al. 2001), and (II) enteral salt loading may induce intra-arterial UGN release from the intestinal mucosa (to stimulate renal natriuresis) (Lorenz et al. 2003). Collectively, these data suggest that GN and UGN are released from distinct subsets of brush cells upon acidic or hypertonic stimulation, and that their release serves to regulate luminal pH and osmolarity.

We observed expression of *Gucy2c*, but not of *Guca2a* or *Guca2b*, in the duodenal glands. This GCC pool may be targeted by GN and/or UGN released into the duodenal lumen (although diffusion of peptides into the glands is likely to be opposed by secretory fluid movement) and/or by systemic GN and UGN. In support of the latter option, intra-arterial GN or UGN has been shown to trigger duodenal bicarbonate secretion (Bengtsson et al. 2007). Indeed, systemic UGN has been shown to elicit additional GCC-dependent effects, notably in neuroendocrine tissues (Valentino et al. 2011; Gong et al. 2011). In further support of such actions in the gut, it was shown that contraluminal administration of STa to duodenal enteroids provoked GCC-mediated inhibition of Na^+^, H^+^ antiport activity, strongly suggesting that such oligopeptides are translocated across the epithelium, although the presence of GCC at the basolateral aspect of polarized intestinal epithelial cells cannot be excluded completely (Foulke-Abel et al. 2016).

Distal from the duodenum, prominent *Guca2a* and *Guca2b* expression is found in the columnar (absorptive) cells of the villus. Strong expression of *Guca2a* and *Guca2b* in the villus has been observed before in mice, but not in rat or guinea pig (Cetin et al. 1994; Steinbrecher et al. 2002; Perkins et al. 1997; De Sauvage et al. 1992; Wang et al. 2012b). Thus, the increase in total expression of *Guca2a* and *Guca2b* distal from the duodenum can be mainly attributed to expression initiated in enterocytes. This expression pattern is similar to that of the transcription factor *Cdx2*, suggesting that this, or other intestine-specific transcription factors, controls *Guca2a* and *Guca2b* expression in enterocytes (Fang et al. 2006). GN and/or UGN released in this region is unlikely to enter the crypts, which contain most of the CFTR-mediated anion-secretory capacity, and a function in the villus compartment seems indicated. Apart from activating a subpool of CFTR, GN and UGN may limit sodium absorption through NHE3, which is highly expressed in mature enterocytes and is inhibited through cGMP-dependent protein phosphorylation (Foulke-Abel et al. 2016). Assuming that peptides released at the surface of the villi would diffuse more rapidly into the bulk luminal content than those secreted in the crypts, local GCC activity and ion transport may simply be controlled by the level to which its ligands are diluted upon their release; GCC stimulation would peak at a low level of hydration, when a high concentration of its ligands is maintained at the epithelial surface. This may constitute a relatively straightforward mechanism to maintain fluid homeostasis, which does not so much rely on a stimulus-controlled release of GN and UGN, but on a continual secretion from columnar cells in the villus. It is of interest that the combined *Guca2a* and *Guca2b* expression in this compartment is particularly high in the distal small intestine, a region which appears especially prone to dehydration and obstruction caused by GCC deficiency (Romi et al. 2012).

In the colon, strong *Guca2a* expression was found in cells positioned at the lateral sides of the crypts. At the base of the crypts, expression was markedly lower and confined to a Paneth-like cell type (Rothenberg et al. 2012). The distal colon in particular contains high numbers of goblet cells, and these also appeared to express *Guca2a*. As in the small intestine, GN release from goblet cells may play a role in mucin expansion. *Guca2b* expression was low in colon, but was retained in solitary cells in the crypts. Strikingly, high *Guca2a* and *Guca2b* expression was found in those parts of the epithelium that were in contact with lymphoid tissue. This particular expression pattern has been observed before for *Guca2b* in the ileocecal region of mice (Whitaker et al. 1997). Because the follicle-associated epithelium also shows strong expression of genes encoding specific ion channels, i.e., *Cftr*, *Clca2*, *Kcnj15,* and *Kcna1* (Kobayashi et al. 2012), and we also found weak expression of *Gucy2c* in this region, it is conceivable that GN and UGN regulate ion transport across the follicle-associated epithelium through autocrine signaling.

In summary, the present study charts *Guca2a*, *Guca2b*, and *Gucy2c* expression along the entire murine intestinal tract. Whereas *Gucy2c* expression is relatively uniform along the rostrocaudal and crypt–villus axes, strong regional differences exist in the expression of its ligands. These differences mostly reflect varying levels of expression in the upper regions of the epithelium. *Gucy2c* expression was found in the duodenal glands, suggesting it serves a role in UGN/GN-dependent duodenal bicarbonate secretion. Further, we show distinct expression of *Guca2a* and *Guca2b* in Paneth cells and brush cells, suggesting that GN and UGN play a role in chemoreception, stem cell proliferation, and host defense.

## Electronic supplementary material

Below is the link to the electronic supplementary material.

Supplementary material 1 (DOCX 16 kb)

Supplementary material 2 (JPEG 508 kb)
