# Supplementary material for: Guanylin and uroguanylin are produced by mouse intestinal epithelial cells of columnar and secretory lineage
Source: Histochem Cell Biol. 2016 May 31;146(4):445–55. doi: 10.1007/s00418-016-1453-4 (PMC5037145; doi:10.1007/s00418-016-1453-4)
Supplement: Supplementary file 1 — Supplementary material 1 (DOCX 16 kb) [file 418_2016_1453_MOESM1_ESM.docx]

**Supplementary data**

**Guanylin and uroguanylin are produced by mouse intestinal epithelial cells of columnar and secretory lineage**

Pauline T. Ikpa, Hein F.B.M. Sleddens, Kris A. Steinbrecher, Maikel P. Peppelenbosch, Hugo R. de Jonge, Ron Smits, Marcel J.C. Bijvelds

Corresponding author

M.J.C. Bijvelds, PhD

Erasmus MC University Medical Center

Dept of Gastroenterology and Hepatology

PO Box 2040

3000CA Rotterdam

The Netherlands

Tel: +31 10 7031492 / Fax: +31 10 7032793

Email: [m.bijvelds@erasmusmc.nl](mailto:m.bijvelds@erasmusmc.nl)

**Table S1** Sequence of the primers used for qPCR.

| **Protein** | **Gene** | **Forward primer** | **Reverse Primer** |
| --- | --- | --- | --- |
| GN | *Guca2a* | GATCCTGCAGAGGCTAGAGG | AAGGCAAGCGATGTCACTCT |
| UGN | *Guca2b* | AGGAGATGTCCAATCCCCAG | ACAGTTCACATTCGTCGGTGG |
| GCC | *Gucy2c* | TGTGAACGCGACTTTCATCTAC | GCAGCCCATCTTATGATCTCTTG |
| GAPDH | *Gapdh* | TTCCAGTATGACTCCACTCACGG | TGAAGACACCAGTAGACTCCACGAC |

**Figure S1**

RNAscope analysis of *Guca2a* transcript in jejunal tissue of normal (a) and *Guca2a* null (b) mice. In control mice, *Guca2a* staining matched the pattern shown in Figure 3, but in *Guca2a* null mice, only sparse punctuate staining was found in the nuclei (indicating hybridization with DNA), and cytoplasmic region (indicating weak hybridization with truncated *Guca2a* transcripts).
