# Supplementary figures and images for: Guanylin and uroguanylin are produced by mouse intestinal epithelial cells of columnar and secretory lineage
Source: Histochem Cell Biol. 2016 May 31;146(4):445–55. doi: 10.1007/s00418-016-1453-4 (PMC5037145; doi:10.1007/s00418-016-1453-4)

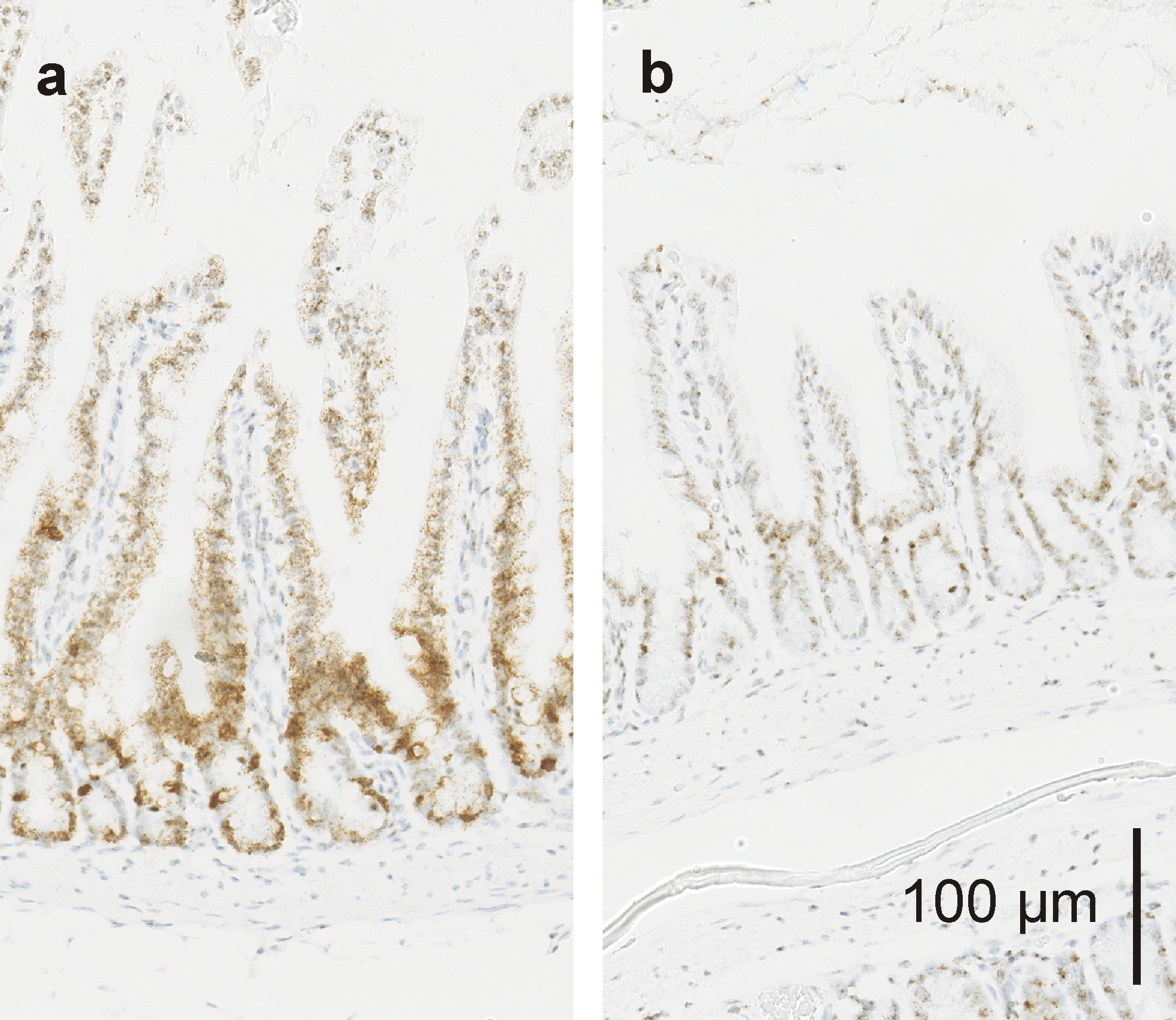

Supplement: Supplementary file 2 — Supplementary material 2 (JPEG 508 kb) [file 418_2016_1453_MOESM2_ESM.jpg]
